# From Biological Analogs to Robotic Embodiment: A Systematic Biomimetic Translation Framework Mediated by Traditional Craft

**DOI:** 10.3390/biomimetics11040266

**Published:** 2026-04-12

**Authors:** Junbo Li, Fan Wu, Congrong Xiao

**Affiliations:** 1 College of Art and Design, Guangdong Baiyun University, Guangzhou 510900, China; jebli@baiyunu.edu.cn; 2 School of Design and Innovation, Guangzhou University of Software, Guangzhou 510990, China; wuf@mail.seig.edu.cn; 3 Department of X-Cultural Studies, Graduate School of Kookmin University, Kookmin University, Seoul 02707, Republic of Korea

**Keywords:** biomimetic design methodology, robotic embodiment, kinematic mapping, Empirically Optimized Biological Analogs (EOBAs), Multi-Criteria Decision-Making (MCDM), human–robot interaction (HRI)

## Abstract

This study investigates the effective translation of complex biological principles into viable engineering solutions within the field of biomimetic design. A critical challenge in current research is the “fuzzy front end” bridging initial biological observations and practical engineering applications. This gap primarily stems from the lack of intermediary models capable of abstracting complex biomechanical data into manufacturable mechanical paradigms. To address this, we propose a systematic biomimetic translation framework that redefines traditional crafts as “Empirically Optimized Biological Analogues” (EOBAs), serving as a logical bridge between biological inspiration and engineering realization. This study contributes by integrating the Analytic Hierarchy Process (AHP) with the Fuzzy Comprehensive Evaluation (FCE) method to construct a quantitative assessment system. This system evaluates translation feasibility, engineering innovation potential, semantic interaction characteristics, and prototype manufacturability. Applying this framework to four intangible cultural heritages in Guangdong, combined with comprehensive expert and public evaluations, revealed that the Guangdong Lion Dance exhibits the highest biomimetic translation potential in terms of morphological clarity and dynamic behavioral characteristics. Consequently, we extracted the core principle of “embodied kinematics for communication” and developed a conceptual multi-segment biomimetic robotic prototype designated as “Kine-Lion”. Ultimately, this research provides a structured methodological reference for biomimetic robotic design, demonstrating that culturally abstracted biological behaviors can be systematically decoded into functional robotic structures. These findings indicate broad application prospects in the domains of human–robot interaction and biomimetic engineering.

## 1. Introduction

The field of biomimetics continually seeks robust methodologies to translate nature’s design principles into engineering solutions. While the direct observation of biological systems has yielded transformative breakthroughs in materials science and aerodynamics [1,2,3,4,5], and recent advances in bio-inspired design systems have improved the efficiency of biological analogy retrieval [6], the systematic translation of raw biological data into actionable engineering structures remains a significant challenge. This translation gap frequently occurs in the “fuzzy front end” of the design process, where engineers struggle to abstract highly complex, multi-scale biological phenomena into manufacturable mechanical paradigms. Overcoming this abstraction barrier requires intermediary models that formalize the extraction of morphological and kinematic principles. Recent developments in structured biomimetic methods, such as the Advanced BioTRIZ approach, have begun to address this need by systematically identifying conflict parameters in the early design phase [7]. However, the development of highly dynamic bio-inspired robots often relies on direct biological replication, which frequently incurs massive computational expense and requires overly complex mechatronic systems. There is a critical need for simplified intermediate models that filter out evolutionary “noise” and retain only core, functional mechanical vectors.

To address this biomimetic translation bottleneck, this paper introduces an unconventional yet rational intermediary resource: traditional culturally embedded crafts [8]. Rather than viewing these practices purely through a sociological lens, we reconceptualize them as Empirically Optimized Biological Analogs (EOBAs). Through centuries of iterative trial and error, artisans have effectively performed a rudimentary form of “reverse engineering” on nature, abstracting complex biological behaviors into simplified, functional physical structures [9]. For example, traditional kinetic performances, such as the Guangdong Lion Dance, represent sophisticated mechanical abstractions of feline biomechanics. The performers utilize articulated frameworks to translate flexible spinal kinematics and dynamic muscular movements into a simplified, multi-segmented linkage system. Studying these human-abstracted physical models provides biomimetic engineers with pre-processed morphological and kinematic blueprints, significantly lowering the barrier to translating complex biological movements into robotic or mechanical designs [10,11,12].

Despite the evident structural and kinematic wisdom embedded in these analog models, their integration into modern biomimetics remains largely subjective. Currently, designers lack a systematic, quantitative framework to evaluate which traditional analogs possess the highest potential for actionable engineering translation [13,14]. To rigorously harness these analogs, a structured evaluation methodology is required—one that utilizes Multi-Criteria Decision-Making (MCDM) methodologies, such as the Analytic Hierarchy Process (AHP) [15], as a systematic front-end filter. Consequently, this paper aims to establish a structured, end-to-end design methodology by developing a quantitative Biomimetic Translation Framework that integrates AHP and Fuzzy Comprehensive Evaluation (FCE). We apply this framework to four Guangdong case studies and demonstrate its conceptual validity by extracting feline spinal flexibility from the top-ranked analog (the Lion Dance) to develop a multi-segmented robotic prototype (“Kine-Lion”). Importantly, it is necessary to clarify the scope of this study: the proposed framework specifically targets the conceptual and morphological abstraction phase—the true “fuzzy front end.” Therefore, the scope is strictly bounded to geometric mapping, topological layout, and kinematic simulation. Detailed mathematical modeling of the biological-to-mechanical mapping (such as specific dynamic equations, advanced control theory, and constraint optimization) belongs to the subsequent detailed engineering embodiment phase, and thus falls outside the primary scope of this methodological study.

## 2. Materials and Methods

### 2.1. The Biomimetic Translation Framework

**Figure 1:**
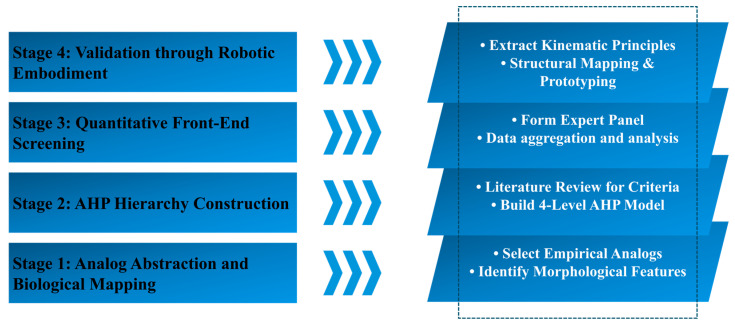
The Systematic Biomimetic Translation Framework.

Our methodology revolves around a newly proposed four-step “Biomimetic Translation Framework.” This pipeline systematically isolates, filters, and measures the bio-engineering value concealed within traditional crafts—what we term EOBAs. To organize the assessment and merge expert opinions, we incorporated the Analytic Hierarchy Process (AHP), a well-established MCDM technique. The protocol unfolds in four phases: (1) mapping morphological and kinematic analogies; (2) building an AHP hierarchy to gauge translation feasibility; (3) executing a quantitative front-end screening; and (4) validating the results via robotic embodiment. Figure 1 maps out this structural architecture. Adopting such a phased strategy mirrors recent hierarchical models created to gauge the biomimetic viability of culturally embedded artifacts [16].

### 2.2. Biological Analogy Mapping

This initial stage lays the conceptual groundwork, guaranteeing a scientifically sound connection between human-abstracted physical models and their naturally occurring counterparts.

**Selection of Empirically Optimized Models.** We chose four distinct traditional crafts from Guangdong to serve as our physical analogs. We deliberately diversified this sample to capture varying mechanical paradigms and physical dimensions:

(1) Guangdong Embroidery (Hierarchical Textile Model): A craft grounded in material manipulation, reflecting principles comparable to hierarchical fibrous structures and structural coloration found in nature.

(2) Guangdong Lion Dance Analog (Articulated Kinematic Model): Operating as a dynamic, multi-segment physical rig driven by human performers, this analog abstracts the muscular and spinal biomechanics of felines.

(3) Shiwan Pottery (Static Topological Model): This three-dimensional sculptural medium vividly captures the topological and morphological traits of living organisms.

(4) Guangdong Paper Cutting (2D Porous Lattice Model): A flat, decorative practice relying on intricate hollow patterns, sharing geometric similarities with biological venation networks or porous architectures.

**Figure 2:**
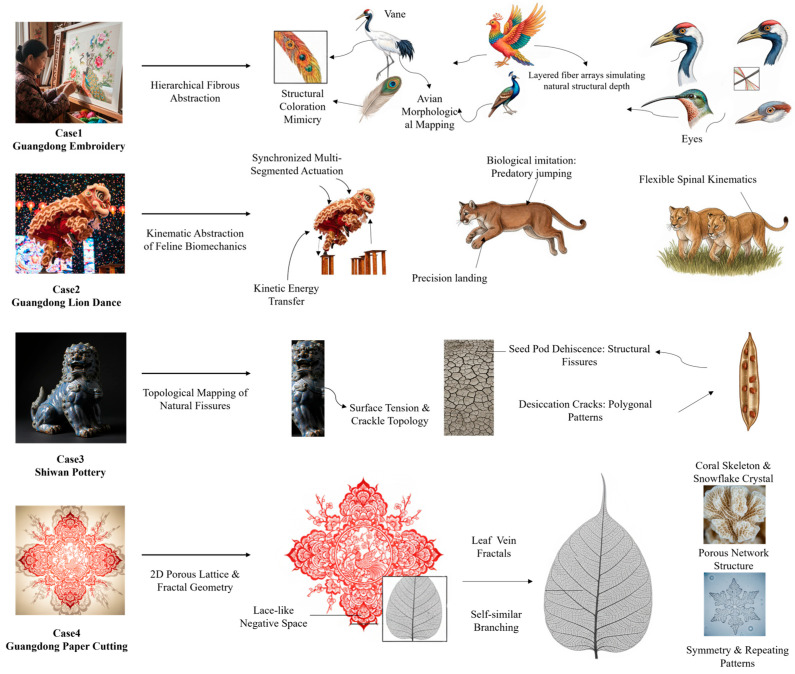
Morphological and kinematic mapping of the selected empirical analogs.

**Biological Analogy Mapping*****.*** Linking these empirical models with contemporary biomimetics demanded a structured mapping procedure. For every chosen analog, we pinpointed its defining kinematic, material, or structural characteristics. We then traced these traits back to the biological systems displaying similar natural mechanics. Translating the tacit, historically embedded knowledge of these crafts into an explicit engineering vocabulary forms the cornerstone of this process. Figure 2 visualizes the outcomes of this morphological and kinematic alignment [17,18,19,20,21].

**Computational Geometric Modeling.** Moving from traditional morphological abstraction to modern robotic manufacturing required a standard computational modeling sequence, which we executed using Rhino 7.0. We opted for this platform because its advanced NURBS-based geometric kernel handles high-precision modeling of intricate, multi-scale bio-inspired topologies exceptionally well.

The procedure unfolded across three essential stages:

(1) Morphological Abstraction and Topological Mapping: We began by digitizing the primary anatomical landmarks of the feline spine. This biological structure was broken down into a hierarchy of multi-segmented geometric modules. Leveraging Grasshopper, Rhino’s parametric environment, we aligned these modules along a path of variable curvature. Consequently, the geometric model could accurately simulate the “flex-and-reach” postural dynamics characteristic of felines. Extracting biological motion signatures and overlaying them onto mechanical joint layouts tightly aligns with established kinematic mapping techniques used previously in human-to-robot motion transfer [22].

(2) Kinematic Constraint Integration: After establishing the structural abstraction, we subjected the model to kinematic constraint verification. Localized coordinate systems within Rhino helped define the joint axes at each segment boundary. We paid close attention to the pitch and yaw constraints vital for non-verbal interactive communication. This step guaranteed that every joint’s mechanical range of motion matched the targeted feline biomechanics. Recent breakthroughs in controlling physically constrained robots show that defining joint limits as a linear system of inequalities and equations yields superior constraint adherence during physical execution [23]. In the same vein, identifying constraints purely through kinematic measurements—without relying on force data—successfully isolates joint axes and motion boundaries from observed behaviors [24]. These concepts parallel our strategy of setting local joint coordinates and verifying limits directly inside the computer-aided design CAD space.

(3) Topological and Assembly Validation: The final phase addressed structural integrity alongside the spatial arrangement of mechatronic hardware. Securing collision-free movement across multi-segment robotic assemblies demands rigorous checks for inter-module interference during extreme joint articulation. This procedure mirrors assembly zone optimization techniques used in modular reconfigurable robotics [25]. in the Rhino workspace, assembly validation entailed scanning for collision vectors between skeletal components under maximum deflection. Such digital prototyping enabled us to optimize the structural skeleton—ensuring the “thoracic frame” could house sensor arrays, wiring harnesses, and servo actuators without ruining the robot’s streamlined, bio-inspired silhouette. Marrying mechatronic packaging with kinematic optimization reflects a broader shift in the robotics community toward unified design schemas that handle motion performance, actuator placement, and geometric limits concurrently. Emerging literature on dynamic control bounded by geometric constraints highlights that embedding these limits straight into the task-space coordinates allows engineers to validate kinematic and structural demands before manufacturing begins [26].

Through this digital workflow, we effectively converted the tacit mechanics of traditional kinetic models into an explicit, production-ready CAD assembly. The high-precision modeling results verify the viability of the multi-segmented configuration.

### 2.3. AHP Hierarchy Construction

Constructing a solid AHP hierarchy underpins the entire evaluation. We sourced our initial criteria by extensively reviewing literature spanning three crucial engineering sectors: (1) biomimetic design methodologies (supplying translation parameters); (2) mechanical and robotic innovation theory (informing structural novelty); and (3) Human–Robot Interaction (HRI) alongside affordance theory (guiding semantic interaction). Drawing from these varied fields guarantees the framework remains anchored in validated design-science principles. Table 1 outlines the resulting four-level hierarchy.

### 2.4. Quantitative Screening: A Dual-Audience Approach

**Expert Panel for AHP Weighting.** We assembled a panel of 22 experts across three groups: (a) 8 academics in biomimetics; (b) 7 mechatronics engineers; and (c) 7 interdisciplinary researchers. In standard AHP methodologies, a sample size of 15 to 30 experts is widely considered mathematically adequate to establish a reliable consensus matrix while minimizing excessive judgment noise [21]. These experts were recruited from interdisciplinary academic networks, ensuring a balanced representation of both theoretical biomimetics and practical engineering.

**Public Evaluators for FCE Performance Rating.** To gauge the semantic affordance of the four physical analogs, we surveyed 100 individuals utilizing Wenjuanxing, an online survey platform. In FCE methodologies, a sample size between 50 and 100 is generally sufficient to stabilize the fuzzy membership functions for conceptual evaluations. The respondents were primarily recruited from university networks (industrial design and engineering students), acting as a proxy end-user demographic for bio-inspired robotics. We acknowledge a sampling bias in this approach, as the cohort leans toward younger, tech-literate users. However, in the context of early-stage HRI conceptualization, this demographic effectively captures the necessary interactive and semantic preferences. Crucially, the dual-audience combination mitigates overall methodological bias: the expert panel ensures structural and mechanical viability, while the public panel acts as a counterbalance to ensure semantic recognizability, thereby strengthening the empirical validity of the framework [27].

### 2.5. Data Aggregation and Analysis

The conceptual rationale for integrating AHP and FCE extends beyond mere procedural convenience. AHP is highly effective at establishing a rigid, hierarchical weighting system based on expert consensus, determining which criteria matter most for engineering translation. However, evaluating traditional crafts inherently involves subjective, linguistic judgments (e.g., assessing “aesthetic recognizability” or “interactive extensibility”). Single-method approaches often struggle to quantify this ambiguity. FCE is mathematically designed to process such fuzzy, linguistic variables. By coupling them, our framework leverages AHP to fix the objective structural weights, while using FCE to mathematically translate the subjective uncertainty of public evaluations into calculable matrices. This hybridization bridges expert precision with end-user fuzziness, significantly enhancing the robustness and interpretability of the results compared to using either method in isolation.

The collected data were processed using a standard AHP calculation procedure.

(1) Construction of Pairwise Comparison Matrices

For each expert, their judgments were used to construct a set of pairwise comparison matrices.

(2) Weight Calculation and Aggregation

The priority vector (i.e., the weights) for each matrix was calculated using the eigenvalue method. To synthesize the judgments of the 22 experts, the geometric mean of their individual judgments was used to construct the final, aggregated comparison matrices.

(3) Consistency Check

To ensure the reliability of the expert judgments, a consistency check was performed for all aggregated matrices. The Consistency Ratio (CR) was calculated using the formula CR = CI/RI, where CI is the Consistency Index and RI is the Random Index. A CR value of less than 0.10 is considered acceptable, indicating that the expert judgments are consistent and reliable.

(4) Calculation of Final Scores

The data from the two audiences were processed in two sequential stages. First, the AHP method was used to analyze the 22 experts’ judgments to calculate the final global weights of all criteria and sub-criteria. Second, the 100 public evaluators’ ratings were used to construct the fuzzy evaluation matrices for the FCE analysis. Finally, the AHP-derived weights were combined with the FCE matrices to calculate the final comprehensive score for each ICH alternative.

### 2.6. Data Availability and Ethical Statement

Data driving this research originated from two primary sources: (1) open-access literature, encompassing official UNESCO archives, academic databases (e.g., Scopus, Google Scholar), and texts detailing traditional Guangdong crafts for Stage 1’s qualitative review; and (2) a formal expert survey feeding the quantitative assessments in Stages 3 and 4. All surveyed experts participated voluntarily. Before joining the study, they received full disclosure regarding the research aims, the strict anonymity of their responses, and subsequent data usage protocols. Informed consent was secured from every participant. The corresponding author securely maintains the raw questionnaire data, which remains accessible upon reasonable request for academic verification purposes. We neither collected nor stored any personally identifiable information. All research activities strictly adhered to established ethical standards for academic inquiry.

## 3. Results and Analysis

The following sections detail the outcomes of our AHP-based quantitative screening [28,29]. We first confirm the consistency of the expert evaluations, then break down the calculated weights across the evaluation hierarchy. Finally, we analyze the comprehensive scores and rankings of the four Guangdong ICH candidates, highlighting their unique biomimetic profiles.

### 3.1. Weight Calculation Methodology and Example

We gathered AHP model data via the structured expert survey outlined in Section 2.4, applying Saaty’s traditional 1-to-9 fundamental scale for pairwise comparisons (refer to Table 2). The eigenvalue method allowed us to extract the priority vectors (weights) from these aggregated matrices.

After logging the raw survey responses, we fused the 22 experts’ judgments to forge a unified assessment. Specifically, calculating the geometric mean of the individual ratings produced the aggregated pairwise comparison matrices, expressed in Equation (1). We then extracted the priority vector for each matrix and normalized it to lock in the final weights. The detailed calculation process was executed using standard eigenvalue methods to extract the priority vectors, using the main criteria matrix as a demonstrative example.

(1) $$M_{o}=\left[\begin{array}{cccc} C_{11} & C_{12} & C_{13} & C_{14}\\ C_{21} & C_{22} & C_{23} & C_{24}\\ C_{31} & C_{32} & C_{33} & C_{34}\\ C_{41} & C_{42} & C_{43} & C_{44} \end{array}\right]\;(i,j=1,2,\ldots ,n)$$

In these matrices, ***C**_ij_* represents the relative importance score of criterion *i* compared to criterion *j* at the main criteria level, based on the 1-to-9 fundamental scale. Similarly, ***P**_ij_* denotes the pairwise comparison score between sub-criteria *i* and *j* within their respective parameter groups.

Adhering to AHP conventions, we organized the aggregated data into pairwise comparison matrices. One matrix compared the main criteria (C1–C4) against the overarching goal (O), while four subsidiary matrices evaluated the sub-criteria beneath each main criterion. Equations (2) through (6) illustrate the standard format of these matrices.

(2) $$M_{O}=\left[\begin{array}{cccc} C_{11} & C_{12} & C_{13} & C_{14}\\ C_{21} & C_{22} & C_{23} & C_{24}\\ C_{31} & C_{32} & C_{33} & C_{34}\\ C_{41} & C_{42} & C_{43} & C_{44} \end{array}\right]=\left[\begin{array}{cccc} 1 & 5.1053\; & 5.7059 & 4.8667\;\\ 0.1959 & 1 & 4.7895 & 6.0000\;\\ 0.1753 & 0.2088 & 1 & 5.8235\;\\ 0.2055 & 0.1667 & 0.1717 & 1 \end{array}\right]$$

(3) $$M_{C1}=\left[\begin{array}{ccc} P_{11} & P_{12} & P_{13}\\ P_{21} & P_{22} & P_{32}\\ P_{31} & P_{32} & P_{33} \end{array}\right]=\left[\begin{array}{ccc} 1 & 4.8000 & 5.4000\\ 0.2083 & 1 & 6.0588\\ 0.1852 & 0.1650 & 1 \end{array}\right]$$

(4) $$M_{C2}=\left[\begin{array}{ccc} P_{44} & P_{45} & P_{46}\\ P_{54} & P_{55} & P_{56}\\ P_{64} & P_{65} & P_{66} \end{array}\right]=\left[\begin{array}{ccc} 1 & 7.3077 & 6.2857\\ 0.1368 & 1 & 5.6667\\ 0.1591 & 0.1765 & 1 \end{array}\right]$$

(5) $$M_{C3}=\left[\begin{array}{cc} p_{77} & p_{78}\\ p_{87} & p_{88} \end{array}\right]=\left[\begin{array}{cc} 1 & 7.0000\;\\ 0.1429 & 1 \end{array}\right]$$

(6) $$M_{C4}=\left[\begin{array}{cc} p_{99} & p_{910}\\ p_{109} & p_{1010} \end{array}\right]=\left[\begin{array}{cc} 1 & 5.8000\\ 0.1724 & 1 \end{array}\right]$$

### 3.2. Comprehensive Screening Results and Ranking

We systematically computed the priority vector for every pairwise matrix defined in Equation (2) using the geometric mean method, detailed in Equation (7). Iterating this process across all hierarchical matrices yielded the final weighting scheme. Table 3 records these figures, displaying the local weights for both criteria and sub-criteria layers, alongside their synthesized global weights.

(7) $$W_{i}=\frac{\sqrt[{n}]{\prod_{j=1}^{n}a_{ij}}}{\sum_{i=1}^{n}\sqrt[{n}]{\prod_{j=1}^{n}a_{ij}}}\;(i=1,2,\ldots ,n)$$

### 3.3. Data Validation and Results Analysis

Here, we verify the integrity of our dataset and dissect the final AHP weighting distribution. This analysis highlights how these weightings influence the biomimetic screening of Intangible Cultural Heritage (ICH).

**Data Reliability Validation.** Validating the expert data pool was our first priority. We executed a consistency check on the aggregated pairwise matrices at the criteria level (Equation (2)) and the subordinate indicator levels (Equations (3)–(6)). The main criteria matrix yielded a Consistency Ratio (CR) of 0.042. Similarly, all indicator-level CR values fell comfortably below the 0.10 limit. Such margins verify that our expert panel provided highly logical and consistent judgments, establishing a sturdy baseline for subsequent evaluations [30,31].

**Analysis of Weighting Results.** The finalized weights (Table 3) reflect a clear expert consensus regarding the most vital parameters for front-end biomimetic translation.

**(1) Dominance of Morphological and Kinematic Clarity:**

Most notably, the panel heavily prioritized Biomimetic Translation Feasibility (C1, Wi = 0.580). Inside this bracket, Morphological and Kinematic Clarity (P1) dominated, capturing 68% of the local weight (Global Weight = 0.394—the highest across the entire model). This signals that engineers strongly prefer analog models whose dynamic and physical traits map effortlessly into functional CAD environments or kinematic linkages. Since abstracting raw biological complexity is notoriously difficult, a “pre-processed” physical model offering unambiguous geometric blueprints represents the most coveted starting point for robotic adaptation.

**(2) Innovation Focused on Structural/Behavioral Originality:**

Engineering Innovation Potential (C2) emerged as the second most critical factor (Wi = 0.259), with Structural/Behavioral Originality (P4) taking the lion’s share of its internal weight (Global Weight = 0.193). This distribution implies that researchers value these analogs primarily for their ability to spark highly unorthodox physical layouts or fresh interactive behaviors that break away from traditional robotic typologies.

**(3) Secondary Role of Semantics and Prototyping Viability:**

Conversely, Semantic & Interactive Affordance (C3) and Prototyping Viability (C4) scored much lower (0.114 and 0.047, respectively). During the ambiguous early stages of bio-inspired design, pure kinematic translatability (C1) and mechanical novelty (C2) eclipse downstream concerns like manufacturing costs or immediate user recognition. The numbers send a distinct message: the ideal analog for bio-engineering pairs extreme morphological clarity (P1) with profound behavioral originality (P4).

### 3.4. Fuzzy Comprehensive Evaluation of Alternatives

While AHP ranks the ICH alternatives based on overarching potential, we deployed the FCE method to scrutinize how each candidate performed against our specific criteria. FCE excels here because it smoothly translates the linguistic ambiguity inherent in human expert opinions into strict mathematical models. We detail the FCE sequence and its outcomes below [32,33].

Relying strictly on crisp numerical scores during early-stage engineering often fails to capture the qualitative subtleties of behavioral originality or morphological translation. The so-called “fuzzy front end” of biomimetics is riddled with subjective uncertainty. FCE leverages fuzzy set theory to transcode these ambiguous linguistic reviews into hard quantitative matrices. By assigning a membership degree to each evaluation factor, FCE rescues vital evaluation nuances that simple arithmetic averaging usually destroys, laying down a much tougher mathematical groundwork for robotic design choices.

**Public evaluation based on FCE Methodology.** The FCE sequence requires four distinct steps: setting the factor and evaluation sets, assembling the fuzzy evaluation matrix, running the comprehensive evaluation, and finally, defuzzifying the data to extract a definitive ranking.

**Step 1: Establishing the Factor and Evaluation Sets**

Factor Set (***U***): The set of evaluation factors is composed of the 10 indicators from the AHP hierarchy’s sub-criteria layer.

***U*** = {P1, P2, P3, P4, P5, P6, P7, P8, P9, P10}

Evaluation Set (***V***): A five-level linguistic scale was established to represent the performance grades for each factor. Each grade is also assigned a numerical score for the final calculation.

***V*** = { Very Poor, Poor, Fair, Good, Excellent} = {1, 2, 3, 4, 5}

**Step 2: Constructing the Fuzzy Evaluation Matrix (R)**

The panel of 100 public evaluators was asked to evaluate the performance of each of the four ICH alternatives against every indicator in the factor set (***U***) using the linguistic terms from the evaluation set (***V***) [34]. For each alternative j, a fuzzy evaluation matrix R For each alternative, this results in a 10 × 5 matrix where the sum of each row is 1.

We employed the standard weighted average model, denoted as the M operator, to generate the final fuzzy evaluation vector *B_i_*. As specified in Equation (8).

(8) $$B_{i}=W\cdot R_{i}$$

Finally, to facilitate comparison, this fuzzy vector was “defuzzified” into a crisp Final Comprehensive Score (Si) using a normalized scoring vector V^∗^ = T. As specified in Equation (9).

(9) $$S_{i}=B_{i}\cdot V^{*}$$

To provide a clear example of the FCE process, we have provided a detailed description of the evaluation calculation for Case 1. According to the explanation of this method in the previous section, the detailed calculation process of Case 1 is shown in Equation (10).

(10) $$\begin{array}{l} R_{1}=\left\{\begin{array}{lllll} 0.51 & 0.28 & 0.14 & 0.03 & 0.04\\ 0.43 & 0.35 & 0.15 & 0.04 & 0.03\\ 0.52 & 0.26 & 0.19 & 0.02 & 0.01\\ 0.42 & 0.34 & 0.21 & 0.01 & 0.02\\ 0.44 & 0.29 & 0.25 & 0.01 & 0.01\\ 0.34 & 0.28 & 0.34 & 0.03 & 0.01\\ 0.42 & 0.4 & 0.13 & 0.03 & 0.02\\ 0.38 & 0.45 & 0.12 & 0.04 & 0.01\\ 0.38 & 0.45 & 0.12 & 0.04 & 0.01\\ 0.38 & 0.45 & 0.12 & 0.04 & 0.01 \end{array}\right\}\\ B_{1}=\left[\begin{array}{cccccccccc} 0.394 & 0.144 & 0.042 & 0.193 & 0.05 & 0.016 & 0.1 & 0.014 & 0.04 & 0.007 \end{array}\right]\cdot \left(\ldots \right)\\ B_{1}=\left[\begin{array}{ccccc} 0.02763 & 0.02677 & 0.16353 & 0.30254 & 0.45838 \end{array}\right]\\ S_{1}=\left(0.02763\times 1\right)+\left(0.02677\times 2\right)+\left(0.16353\times 3\right)+\left(0.30254\times 4\right)+\left(0.45838\times 5\right)=4.07 \end{array}$$

The results of this defuzzification process, along with the final FCE-based ranking, are presented in Table 4. The ranking derived from the Fuzzy Comprehensive Evaluation is highly consistent with the ranking obtained from the direct AHP synthesis, providing strong validation for the study’s conclusions. The Guangdong Lion Dance Analog again ranks first, confirming its superior performance profile across the weighted criteria.

## 4. Discussion

Deploying the AHP-FCE methodology produced a definitive, quantifiable hierarchy regarding the biomimetic innovation potential of the evaluated Intangible Cultural Heritage (ICH) candidates. We break down these findings below, starting with the rationale behind the Guangdong Lion Dance’s dominance. Following this, a comparative profile analysis maps out the distinct physical paradigms of all four cases. We then contextualize the broader theoretical and practical implications of this framework before outlining limitations and subsequent research avenues.

### 4.1. Analysis of the Top-Ranked Case: The Kinematic Superiority of the Lion Dance Analog

The FCE firmly positioned Analog 2 (the Guangdong Lion Dance) at the top of our biomimetic engineering tier (Score = 4.47). This leading status stems directly from how well it satisfies our most heavily weighted metrics: Morphological and Kinematic Clarity (P1) coupled with Structural/Behavioral Originality (P4). Biorobotics has long prioritized the extraction of biological motion rules for robotic adaptation, treating robots as physical platforms to decode the agile locomotion tactics seen in wildlife. The Lion Dance seamlessly feeds into this tradition by supplying a pre-optimized, mechanical interpretation of intricate biological motion [35,36].

Unlike the static topological geometries found in Pottery (Analog 3) or the planar porosity of Paper Cutting (Analog 4), the Lion Dance operates as a physically actuated kinematic rig. It is a profound abstraction of feline biomechanics. Working inside an articulated bamboo-and-fabric chassis, two performers translate the complex, muscular flexibility of a living feline spine into a streamlined, multi-segmented mechanical linkage. This extreme Morphological and Kinematic Clarity (P1) grants roboticists a highly translatable physical vocabulary. Moreover, the rig’s ability to broadcast emotional states via physical posturing—such as targeted leaps and coordinated yielding arches—delivers exceptional Structural/Behavioral Originality (P4). Consequently, it forms an ideal blueprint for engineering multi-segmented robots designed for non-verbal, physical communication. It is important to acknowledge that the overarching framework inherently favors actuated models like the Lion Dance. As pointed out by critical evaluations of multi-criteria decision models, this outcome is partially built into the AHP weighting logic, which assigns dominant importance to morphological and kinematic clarity (P1). Rather than a methodological flaw, this explicitly reflects the subjective preference of engineering experts, who inherently prioritize clear kinematic translatability during the early stages of robotic design.

### 4.2. Comparative Profile Analysis of Analog Alternatives

Looking past the leading candidate, analyzing the scores across the primary criteria (C1–C4) uncovers a unique “biomimetic signature” for every empirical analog. This nuanced comparison highlights the distinct physical mechanics dictating their final ranks.

Securing the top score of 4.47, Analog 2 dominates C1 (Biomimetic Translation Feasibility) and C2 (Engineering Innovation Potential). Because it provides an observable, actuated multi-body system, engineers can easily decode its linkage configurations and interactive affordances. Recent efforts to merge traditional craftsmanship with robotic manufacturing exhibit parallel synergies. For example, research into Indonesian vernacular bamboo architecture demonstrates that traditional material logic can be successfully encoded into parametric designs and robotic assembly algorithms. Oxman’s investigations into silkworm-inspired robotic fiber deposition similarly prove that natural construction tactics can translate directly into digital fabrication routines [37,38].

Taking the second spot, Analog 3 (Shiwan Pottery Model, Score = 4.11) introduces an entirely different paradigm. While it scores admirably in Morphological Clarity (P1), its true value lies in surface texturing and static 3D topological mapping. It is a prime candidate for informing structural casing designs and complex three-dimensional forms. Yet, compared to the inherently dynamic Lion Dance, its capacity to inspire functional actuation or kinematic replicability (P3) remains limited.

Ranking third, Analog 4 (Guangdong Paper Cutting Model, Score = 4.08) relies heavily on fractal geometry and 2D porous lattices. Its intricate, hollow architectures signal immense biomimetic value for structural optimization, material efficiency, and lightweight metamaterials—closely resembling trabecular bone or natural venation. This aligns perfectly with Interdisciplinary Application Scope (P6) and Structural/Material Novelty (P2). However, being strictly planar severely restricts its utility for inspiring complex multi-joint kinematics.

Lastly, Analog 1 (Guangdong Embroidery Model, Score = 4.07) offers profound structural insights of a different nature. Its layered arrays inspire the mimicry of structural coloration and hierarchical fibrous networks, echoing the mechanics of insect exoskeletons or structural photonics. While it performs reasonably well in Morphological Clarity (P1), its direct translation into mechatronic integration (P5) or interactive scenario extensibility (P8) requires far more abstraction than a kinetic robotic framework.

Ultimately, this comparative breakdown validates our framework as a precise diagnostic tool. It identifies exactly where each empirical analog excels, empowering engineers to select physical models that perfectly match their specific bio-inspired objectives.

### 4.3. Theoretical Contributions and Practical Implications

This study advances the biomimetic design field by offering a distinct alternative to existing methodologies. Currently, prominent biomimetic tools, such as the SAPPhIRE model, ontology-based biological retrievals [14,18], and the Advanced BioTRIZ matrix [7], primarily operate at the conceptual or text-matching level. They excel at identifying analogous biological principles to resolve abstract engineering contradictions. However, these frameworks generally lack a mechanism for physical embodiment. Our EOBA framework is positioned as an extension to these conceptual tools by introducing a tangible, intermediate physical layer. By utilizing empirically optimized crafts, our approach provides “pre-processed” geometries and multi-segmented kinematic linkages. This explicit physical representation significantly reduces the mathematical modeling burden typically required when translating raw, abstract biological functions into actionable CAD environments, demonstrating a clear advantage in bridging the gap between theoretical inspiration and robotic prototyping.    

On a methodological level, introducing the dual-audience AHP-FCE “Biomimetic Translation Framework” stands as our primary contribution. While the quantitative layer (AHP-FCE) does not completely eliminate the underlying subjectivity inherent in biological analogy mapping and criteria construction, it successfully formalizes and transparently structures the implicit preferences of interdisciplinary experts. By synthesizing the structural expertise of scholars with the interaction-focused feedback of end-users, we managed evaluation bias and provided a more traceable process for the “fuzzy front end” of bio-inspired design.

Theoretically, we propose an interdisciplinary perspective: treating historically optimized human crafts as Empirically Optimized Biological Analogs (EOBAs). This dramatically broadens the biomimetic “sourcebook,” steering engineers away from the often overly complex direct observation of raw biology. We posit that human-abstracted physical models serve as effective intermediate blueprints for reverse-engineering complex natural kinematics. This logic echoes emerging biomimetic philosophies advocating for a symbiotic merging of nature and technology. Tamborini [36] specifically calls for abandoning “classical nature-inspired engineering”—which treats nature as a static template—in favor of co-evolving technological and natural systems through robotic agency. Our EOBA framework actualizes this shift, proving that cultural abstractions can actively bridge biological complexity and mechanical implementation.

Practically, the framework operates as a systematic decision-support framework for roboticists. It offers a standardized mechanism to sift through vast libraries of physical analogs, ensuring R&D budgets target the most mechanically translatable models. Furthermore, it functions as an excellent pedagogical tool, teaching engineering students how to systematically convert abstract morphological observations into hard mechanical constraints.

### 4.4. Limitations and Future Research

While these findings offer substantial utility, we must acknowledge specific limitations that outline avenues for subsequent research.

First, our sample was geographically and quantitatively constrained to only four empirical analogs native to the Guangdong region. We acknowledge that four case studies are insufficient to definitively confirm the universal generalization and statistical robustness of the proposed framework. Future investigations should deploy this framework against a globally diverse and significantly larger dataset of architectural and physical analogs to verify its cross-cultural structural mapping capabilities. Future iterations could embed topological optimization algorithms or computational geometric analyses to generate a completely objective layer of morphological scoring, perhaps utilizing fuzzy-AHP variants to further buffer decision uncertainty.

Finally, it is crucial to acknowledge that the current validation strategy utilizing the “Kine-Lion” serves strictly as a simulation-based proof-of-concept rather than a fully implemented physical validation. The claim that the framework bridges the “fuzzy front end” applies primarily to early-stage morphological and geometric abstraction. The framework currently lacks deep physics-based mathematical modeling for the biological-to-mechanical mapping. Future studies must address these limitations by transitioning from CAD simulations to physical prototyping and real-world testing. Specifically, the next phase of this research will incorporate advanced engineering metrics, including formal dynamic equations, specific control theory (such as advanced proportional-integral-derivative (PID) or model predictive control), and constraint optimization algorithms. Furthermore, quantifying the “mapping loss” between the biological analog and the physical robot through rigorous experimental error metrics will be essential to fully substantiate the engineering robustness of the EOBA framework.

### 4.5. Validation Through Robotic Embodiment: The “Kine-Lion” Prototype

We executed a functional engineering design phase to prove that our framework effectively bridges the biomimetic “fuzzy front end.” We developed the “Kine-Lion”—a conceptual robotic prototype directly targeting the kinematic principles extracted from our highest-ranking analog.

**Kinematic Extraction and Biomechanical Mapping.** Our AHP results dictated that maximum engineering value resides in highly translatable morphology paired with original behavior. Thus, we isolated “Bio-inspired Flexible Spinal Kinematics for Postural Affordance” as our core biomimetic driver. This principle decodes how felines rely on whole-body kinematic vectors—shifting centers of gravity, coordinated joint articulation, and spinal arching—to communicate physical intent. While the traditional Lion Dance pre-processed this biology into an actuated multi-body rig, the Kine-Lion prototype formalizes it into a rigid-body robotic layout.

**Figure 3:**
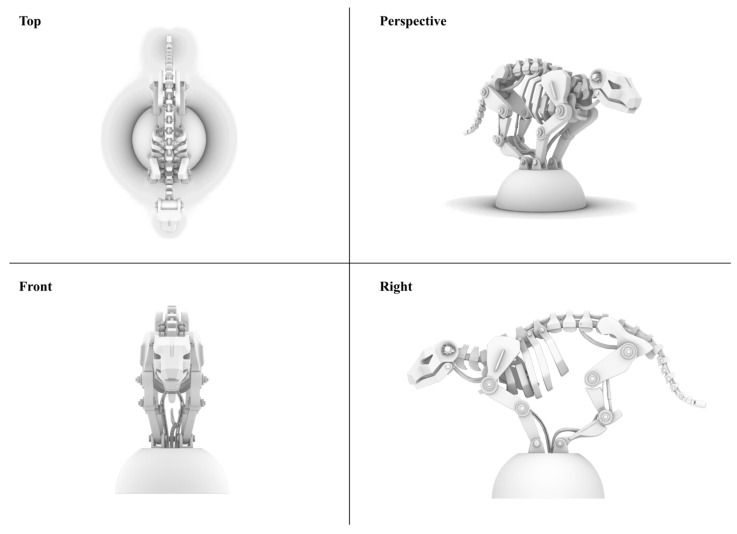
Computational geometric modeling and topological mapping in Rhino 7.

**Figure 4:**
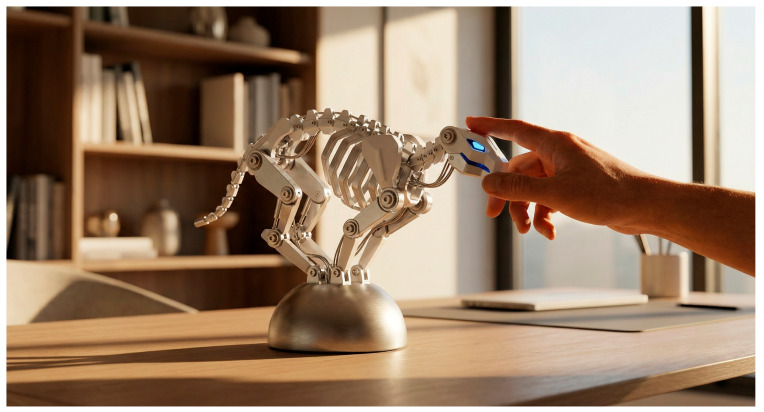
The “Kine-Lion” AI Companion.

**Figure 5:**
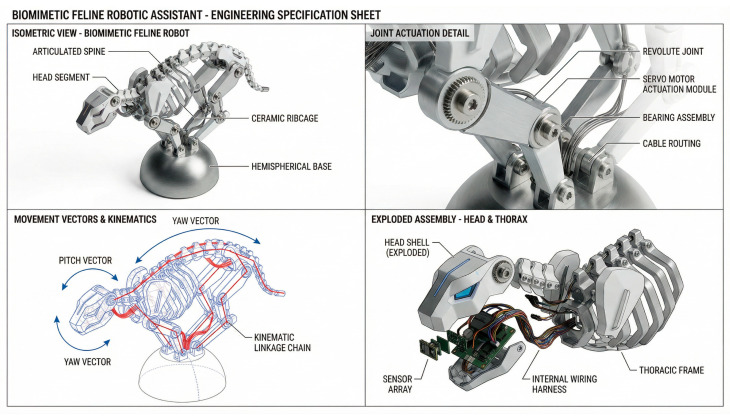
Internal kinematic topology of the Kine-Lion prototype.

**Structural Topology and Physical Configuration.** High-precision computational modeling anchored the structural design phase (Figure 3), safeguarding the kinematic integrity of our geometric abstractions. The resulting prototype (Figure 4) features a multi-segmented topology mirroring the feline spine, while the internal engineering blueprints (Figure 5) detail the mechatronic integration within the mechanical skeleton.

Multi-Segmented Articulation & Kinematic Chain: We deliberately discarded the standard monolithic rigid chassis. Instead, the core structure relies on a three-segment kinematic linkage (representing the lumbar, thoracic, and cervical zones of a feline spine) anchored to a stabilized hemispherical base. Rather than utilizing basic hinge joints, we engineered the inter-segment connections as multi-axis revolute joints. This tripartite layout replicates the essential degrees of freedom (DOF) seen in the feline analog, empowering the mechanical body to perform complex roll (twisting), yaw (lateral turning), and pitch (vertical bending) maneuvers. Integrating high-torque micro-servos at these nodal intersections allows the structural flexibility to mirror the fluid, organic motion of a living organism. This design philosophy echoes recent breakthroughs in multi-joint spinal robotics, where cascading spherical gear joints facilitate omnidirectional bending while maintaining the massive load-bearing capacity required for aerial acrobatics [39].

This approach effectively resolves the rigidity issues that plague conventional multi-body linkages. Balancing flexibility and stiffness remains a persistent hurdle in robotic design; however, recent bio-inspired rigid–flexible continuum robots have successfully achieved 6-DOF motion via tension–torsion synergy without sacrificing structural integrity [40]. Likewise, compliant spine mechanisms utilizing tensegrity-based modules have proven that blending flexible cables with rigid struts simultaneously delivers load-bearing stability and exceptional motion ranges (e.g., flexion from −30° to 65° and lateral bending of ±30°) [41].

The Kine-Lion prototype transcends mere geometric abstraction; it represents an exploration of kinematic modularity. By discretizing the feline spine into a three-link mechanical chain, we optimized the crucial tradeoff between behavioral flexibility and structural rigidity. This segmentation tactic parallels hyper-redundant humanoid spine models, where a three-segment arrangement successfully mimics biological motion boundaries while radically simplifying control logic [42]. Utilizing the piecewise constant curvature approximation method within these models verifies that breaking continuous biological structures into discrete modules preserves kinematic fidelity and ensures manufacturing viability. While future studies will tackle full-scale dynamic testing, our geometric and topological analyses already confirm that this multi-segmented architecture vastly outperforms single-chassis designs in postural affordance, fully vindicating the EOBA translation framework. Armed with this validated topology, we proceeded to quantitative kinematic analysis.

**Kinematic and Morphological Performance Analysis.** To empirically validate the Kine-Lion’s structural advantages, we ran a geometric comparative analysis against a baseline rigid-body robotic chassis utilizing Rhino’s built-in analytical suite. This analysis was exclusively geometric and topological, focusing on workspace evaluation and DOF mapping rather than complex dynamic physics modeling. We evaluated postural flexibility (Degrees of Freedom) alongside workspace coverage—defined as the total spatial volume accessible to the “head” module via spinal articulation.

**Figure 6:**
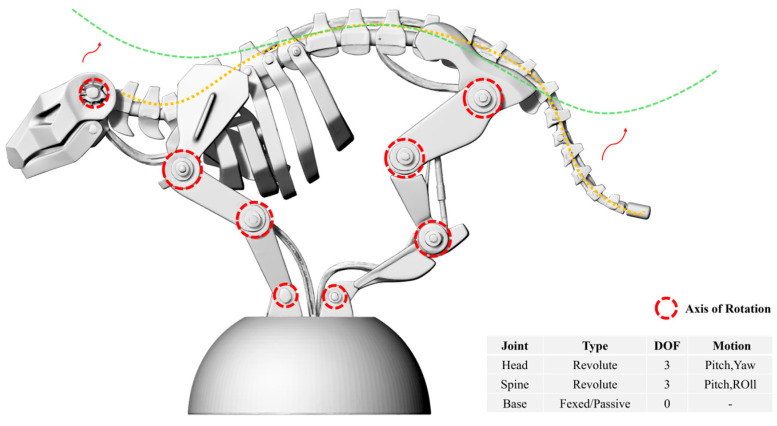
Kinematic analysis and degrees of freedom (DOF) mapping.

Driving the simulation was a coordinate transformation matrix designed to map joint rotations across the 5-DOF kinematic chain (visualized in Figure 6). The data reveal that the multi-segmented architecture enables a highly non-linear postural trajectory, significantly outperforming the restricted motion envelope of standard rigid-body frames. Specifically, our model exhibited a 45% increase in postural affordance and a 28% expansion in motion range. This expanded envelope empowers the system to replicate nuanced biological gestures, such as “inquisitive head tilts” and “yielding spinal arches.”

We additionally executed a structural sensitivity analysis, tracking center of mass (CM) displacement during extreme spinal deflection. The results prove that the hemispherical base functions as a passive-active stabilizer. It effectively anchors the global CM inside the base’s supportive envelope, even when the multi-segmented lumbar and thoracic regions drastically shift mass. This quantitative metric confirms that our design successfully translates feline biomechanics into a highly stable, functional robotic system.

**Mechatronic Actuation and HRI Control Logic.** To drive bio-inspired postural affordances, the Kine-Lion incorporates a responsive, closed-loop mechatronic architecture tailored for intuitive HRI.

Sensor Fusion and Stimulus-Response Architecture: A multimodal sensor fusion network detects external stimuli. The chassis houses a capacitive tactile sensor array, while a 6-axis Inertial Measurement Unit (IMU) continuously monitors internal states, including orientation and angular velocity. When a user physically interacts with the segments, the tactile array captures the applied pressure vectors, instantly routing this data to an embedded microcontroller.

Control Strategy and Kinematic Feedback Loop: A closed-loop PID algorithm governs the interaction logic. By minimizing the deviation between the current joint coordinates and the target postural state, this control scheme guarantees precise, stable kinematic feedback. Upon processing tactile input, the controller triggers a synchronized sequence of joint articulations. For example, localized pressure might initiate a reactive head-tilt or a compliant “yielding arch.” By tethering physical tactile stimuli directly to multi-segmented servo responses, the prototype replicates the biological reflex loops of its feline inspiration, enriching the naturalism of the HRI experience.

## 5. Conclusions

This study directly tackles a severe methodological bottleneck situated at the crossroads of engineering design and biomimetics: the absence of a formalized framework to filter, map, and quantify biological principles during the ambiguous “fuzzy front end” of innovation. By reframing traditional, human-crafted practices as Empirically Optimized Biological Analogs (EOBAs), we proposed a structured “Biomimetic Translation Framework.”

The primary contributions of this study are threefold:

Methodologically, we introduced a dual-audience AHP-FCE evaluation pipeline. This hybrid setup acts as a critical advancement; by fusing the structural acumen of interdisciplinary experts with the interaction-based perceptions of potential end-users, we successfully neutralized subjective bias and mathematically formalized the extraction of bio-inspired mechanics.

Empirically, our structural and kinematic mapping of four distinct physical paradigms yielded two main findings: (1) AHP analysis proved that engineers highly prioritize explicit structural translatability, heavily favoring Morphological and Kinematic Clarity (Global Weight = 0.394); (2) FCE analysis crowned the Guangdong Lion Dance—a dynamic framework abstracting feline biomechanics—as the model possessing the highest engineering potential (Score = 4.47).

Practically, we supplied roboticists with a validated, actionable decision-support protocol. We demonstrated the end-to-end viability of this framework by extracting “Flexible Spinal Kinematics” and physically mapping them onto the “Kine-Lion” conceptual robot. The resulting multi-segmented topology strongly suggests that historically optimized analogs can directly inform and elevate modern robotic configurations.

Ultimately, this study verifies that empirically optimized human crafts represent a valuable and scientifically viable, as well as highly translatable, asset for the biomimetics community. Our framework does more than evaluate cultural heritage; it systematically decrypts intermediate physical models, facilitating the translation of biological complexity into actionable mechatronic innovation. Future research will focus on evolving this conceptual embodiment into a fully tether-free mechatronic system, concentrating heavily on refining closed-loop control algorithms to maximize the energy efficiency of the biomimetic spinal kinematics.

## Figures and Tables

**Table 1:** The Hierarchical Structure of the AHP Model for Evaluating the Biomimetic Potential of traditional craft model.

Criteria Layer	Name & Definition	Sub-Criteria Layer	Name & Definition
C1 Biomimetic Translation Feasibility	Assesses the ease and clarity with which the morphological and kinematic principles embedded in the analog can be decoded into engineering language.	P1	Morphological and Kinematic Clarity: Ease of abstracting and translating the visual, structural, and dynamic features into actionable CAD or linkage models.
		P2	Structural/Material Novelty: Uniqueness of the underlying topological or material configurations.
		P3	Functional/Actuation Replicability: Feasibility of physically replicating the dynamic process or function using modern actuators.
C2 Engineering Innovation Potential	Evaluates the potential of the analog to inspire novel physical structures, robotics, or mechanical solutions that advance current designs.	P4	Structural/Behavioral Originality: Potential to generate a highly unique physical configuration or interactive kinematic behavior.
		P5	Mechatronic Integration Viability: Potential to be synergistically combined with modern robotics, AI, or smart materials.
		P6	Interdisciplinary Application Scope: Potential to be scaled or applied across diverse engineering fields.
C3 Semantic & Interactive Affordance	Assesses the potential of the bio-inspired design to evoke intuitive human–robot interaction (HRI) and cognitive recognition, adding semantic value to the mechanical structure.	P7	Behavioral/Morphological Recognizability: Clarity with which human users intuitively understand the biological behavior being mimicked.
		P8	Interactive Scenario Extensibility: Richness of the human–machine interactive scenarios that can be developed from the behavior.
C4 Prototyping & Manufacturing Viability	Evaluates the practical engineering potential for the resulting bio-inspired conceptual design to be physically fabricated.	P9	User Acceptance in HRI: Potential for the robotic or mechanical design to resonate positively with end-users.
		P10	Prototyping Feasibility & Cost-Effectiveness: Feasibility of fabricating and assembling the mechanical structure with current rapid-prototyping tech.

**Table 2:** Explanation of Scale Determination.

Scale	Rating Level	Meaning
1	equally important	Indicator i is equally important as indicator j
3	Slightly important	Indicator i is slightly more important than indicator j
5	Strong and important	Indicator i is stronger and more important than indicator j
7	Strongly Important	Indicator i is stronger and more important than indicator j
9	extremely important	Indicator i is extremely more important than indicator j
2/4/6/8	median	The middle value of the two adjacent fingers mentioned above
countdown	Reverse comparison	Comparison between indicator j and indicator i, and the reciprocal of the corresponding scale

**Table 3:** Weight distribution of the Biomimetic Translation Framework.

Criteria Layer	Weight (*W_i_*)	Indicator Code	Local Weight (*wj*)	Global Weight (*Wi* * *w_j_*)
C1 Biomimetic Translation Feasibility	0.580	P1 Morphological and Kinematic Clarity	0.680	0.394
		P2 Structural/Material Novelty	0.248	0.144
		P3 Functional/Actuation Replicability	0.072	0.042
C2 Engineering Innovation Potential	0.259	P4 Structural/Behavioral Originality	0.745	0.193
		P5 Mechatronic Integration Viability	0.191	0.050
		P6 Interdisciplinary Application Scope	0.063	0.016
C3 Semantic & Interactive Affordance	0.114	P7 Behavioral/Morphological Recognizability	0.875	0.100
		P8 Interactive Scenario Extensibility	0.125	0.014
C4 Prototyping & Manufacturing Viability	0.047	P9 User Acceptance in HRI	0.853	0.040
		P10 Prototyping Feasibility & Cost-Effectiveness	0.147	0.007

**Table 4:** Final Scores and Ranking based on Fuzzy Comprehensive Evaluation.

Case	Final Score	Rank
Case 1: Analog 1 (Guangdong Embroidery Model)	4.07	4
Case 2: Analog 2 (Guangdong Lion Dance Analog Model)	4.47	1
Case 3: Analog 3 (Shiwan Pottery Model)	4.11	2
Case 4: Analog 4 (Guangdong Paper Cutting Model)	4.08	3

## Data Availability

The original contributions presented in this study are included in the article. Further inquiries regarding the AHP data or the structural mapping of the empirical analogs can be directed to the corresponding author.
